# The Knudsen Layer in Modeling the Heat Transfer at Nanoscale: Bulk and Wall Contributions to the Local Heat Flux

**DOI:** 10.3390/e27050469

**Published:** 2025-04-26

**Authors:** Carmelo Filippo Munafò, Martina Nunziata, Antonio Sellitto

**Affiliations:** 1 Department of Mathematical and Computer Sciences, Physical Sciences and Earth Sciences, University of Messina, Viale F. Stagno d’Alcontres 31, 98166 Messina, Italy; carmelofilippo.munafo@unime.it; 2 Department of Mathematics, University of Salerno, Via Giovanni Paolo II, 132, 84084 Fisciano, Italy; 3 Department of Industrial Engineering, University of Salerno, Via Giovanni Paolo II, 132, 84084 Fisciano, Italy; asellitto@unisa.it

**Keywords:** boundary conditions, phonon–wall interactions, nanoscale heat transfer, Guyer–Krumhansl equation, Knudsen layer

## Abstract

Starting from the observation that the influence of the heat carriers’ boundary scattering on the heat flux is mainly felt in the zone near the system’s boundary, the characteristic dimension of which is of the order of the mean-free path of the heat carriers, in this paper, we introduce the concept of the Knudsen layer in the heat transport at nanoscale and regard the local heat flux as the final resultant of two different contributions: the bulk heat flux and the wall heat flux. In the framework of phonon hydrodynamics, we therefore, here, derive a theoretical model in agreement with the second law of thermodynamics that accounts for those two contributions. In steady states, we then predict both how the local heat flux behaves and how the thermal conductivity depends on the characteristic dimension of the system. This analysis is performed both in the case of a nanolayer and in the case of a nanowire.

## 1. Introduction

A better understanding and modeling of heat transfer at nanoscale are two desirable goals for the optimal thermal management of electronic, optical and optoelectronic devices, as well as for the design of new materials, which could display enhanced thermal-transport properties for energy conversion and utilization.

It is well recognized by now that the heat-transfer phenomenon at the nanoscale distinctly differs from the one at the macroscale [1]: several advances performed in the research on nanoscale heat transfer, in fact, have pointed out a lot of phenomena, wherein the Fourier’s law (which linearly relates the heat flux vector q to the temperature gradient ∇θ by means of the thermal conductivity λ) fails; namely, the well-known constitutive relation q=−λ∇θ is no longer able to adequately describe a lot of current experimental evidence. In the last few decades, therefore, there has been a surge of enhanced theories of heat transport at nanoscale, which encompass the Fourier’s law only as a special case [2,3,4,5,6,7,8,9,10]. Among those theories, each of them characterized by its own peculiar approach [5,11,12,13,14], the one based upon the macroscopic method of the phonon hydrodynamics [15,16,17,18] is worthy of being considered because it allows for depicting in a clear and intuitive way the physics of phonons (i.e., the main heat carriers in non-metallic materials as graphene and silicene) by considering them as particles of a rarefied gas, which, moving throughout the medium owing to the temperature gradient, yield the propagation of the heat flux at nanoscale [5,16,19].

Phonon hydrodynamics also allows for a refined mathematical analysis of the heat transfer at nanoscale, especially if one is wondering which is the correct way of assigning boundary conditions (BCs). To this end, we note that BCs should mathematically depict the interactions between the heat carriers and the lateral walls [20,21]. The consequences of those interactions on the actual value of the heat flux are particularly important at nanoscale and, in principal, mainly felt in a strip near the lateral walls, whose characteristic dimension is of the same order of magnitude of the mean-free path *ℓ* of the heat carriers [16,22,23,24,25,26]. Therein, in fact, the frequency of the collisions of the heat carriers with the lateral walls is much higher than the frequency of any other scattering mechanism appearing in the bulk of the system. By taking inspiration from fluid dynamics, hereafter, we will refer to this strip as the *Knudsen layer*.

Since the characteristic dimensions of many modern nanodevices are comparable with that of the Knudsen layer, it may be interesting to deepen the role of the latter on heat propagation. To this aim, in principle, it could be convenient to start from the assumption that the local heat flux displays both a bulk contribution $q_{b}$ and a wall contribution $q_{w}$. According to this idea, which is also peculiar of Refs. [16,22], for example, in the following, we will hypothesize that $q_{b}$ is principally related to the mechanisms of phonon scattering, characterizing the bulk of the system (i.e., the Umklapp phonon–phonon scattering, the phonon–impurity scattering and the phonon–electron scattering), whereas $q_{w}$ will be meant as the main consequence of the phonon–boundary scattering, which instead characterizes the Knudsen layer. A logical further assumption could be

(1) $$q=q_{b}+q_{w}$$

which can be considered as the constitute relation turning out the actual value of the local heat flux, provided the bulk and wall contributions are known.

After this brief introduction, the paper is organized in four sections. In Section 2, by using considerations in agreement with the second law of thermodynamics, we obtain a theoretical model that could be used to estimate both $q_{b}$ and $q_{w}$. In Section 3, we employ that model in steady states in order to predict both how the local heat flux q, arising from Equation (1), behaves and to obtain an estimation of the effective thermal conductivity in terms of the characteristic dimension of the system. In Section 4, we give some final comments and conclusions.

## 2. The Theoretical Model

By means of macroscopic thermodynamic considerations, in the present section, we obtain a theoretical model that accounts for the possible role played by the Knudsen layer in the heat transfer at nanoscale. To this aim, here, we assume that a generic rigid body $\Omega \subset R^{3}$ at rest, bounded by a piecewise-smooth surface ∂Ω, is crossed by a heat flux q(X,t). The system under analysis, in particular, consists both of a bulk, which is characterized by $q_{b}\left(X,t\right)$, and in the Knudsen layer, which is instead characterized by $q_{w}\left(X,t\right)$. The Knudsen layer is assumed to be superposed to the system’s bulk, ∀X∈Ω and ∀t>0; the former, in particular, fully pervades the latter whenever *ℓ* is larger than the characteristic dimension of Ω. The whole system finally displays its own non-equilibrium temperature θ(X,t), the value of which at the boundary hereafter will be named as *wall temperature* $\theta_{w}\left(X,t\right)$.

In order to only deal with a simple situation, which is, however, physically meaningful, we suppose that ∂Ω does not allow heat exchanges with the external environment; from the mathematical point of view, the consequence of this assumption is that

(2a) $$\begin{array}{cc} q_{b}\cdot \hat{n}=0 & \;\forall \;X\in \partial \Omega ,\;\forall t>0 \end{array}$$

(2b) $$\begin{array}{cc} q_{w}\cdot \hat{n}=0 & \;\forall \;X\in \partial \Omega ,\;\forall t>0 \end{array}$$

with $\hat{n}\left(X\right)$ being the unit normal vector to the boundary ∂Ω, namely, it is normal to the plane tangent to the boundary.

In the present theoretical analysis, which is based upon Extended Irreversible Thermodynamics (EIT) [4,5], we assume that the state (i.e., independent) variables are the internal energy (per unit volume) e(X,t), the bulk heat flux vector $q_{b}\left(X,t\right)$, the wall heat flux vector $q_{w}\left(X,t\right)$, the second-order tensor representing the flux of the bulk heat flux $Q_{b}\left(X,t\right)$ and the second-order tensor representing the flux of the wall heat flux $Q_{w}\left(X,t\right)$. The physical meaning of those second-order tensors can be scrutinized in Ref. [22], for example.

A basic principle of EIT indeed states that all state variables must be characterized by their own evolution equation [4,5], whereas the energy’s balance law naturally leads to

(3) $$\partial_{t}e+\nabla \cdot q=0\;\forall \;X\in \Omega ,\;\forall t>0$$

which can be rightly meant as the evolution equation of the internal energy. The evolution equations of the other state variables are indeed not known a priori, and therefore, they have to be determined; this goal will be pursued here by using a macroscopic approach in agreement with the second law of thermodynamics. In particular, our approach starts from the differential form of the function *s* (mathematically indicating the specific entropy per unit volume), i.e., the so-called Gibbs relation, which reads

(4) $$\begin{array}{cc} ds= & \left(\frac{\partial s}{\partial e}\right)de+\left(\frac{\partial s}{\partial q_{b}}\right)\cdot dq_{b}+\left(\frac{\partial s}{\partial q_{w}}\right)\cdot dq_{w}+\left(\frac{\partial s}{\partial {\overset{o}{Q}}_{b}}\right)\cdot d{\overset{o}{Q}}_{b}+\left(\frac{\partial s}{\partial Q_{b}}\right)dQ_{b}\\ \; & +\left(\frac{\partial s}{\partial {\overset{o}{Q}}_{w}}\right)\cdot d{\overset{o}{Q}}_{w}+\left(\frac{\partial s}{\partial Q_{w}}\right)dQ_{w} \end{array}$$

wherein ${\overset{o}{Q}}_{b}$, ${\overset{o}{Q}}_{w}$ and $Q_{b}$, $Q_{w}$ stand for the deviatoric (traceless) and volumetric parts of the symmetric tensors $Q_{b}$ and $Q_{w}$, respectively.

In order to exploit the thermodynamic consequences of Equation (4), let us now introduce the following constitutive relations

(5a) $$\begin{array}{ccc} \frac{\partial s}{\partial e} & =\frac{1}{\theta } & \forall \;X\in \Omega ,\;\forall \;t\geq 0 \end{array}$$

(5b) $$\begin{array}{ccc} \frac{\partial s}{\partial q_{b}} & =-\left(\frac{\tau_{b}}{\lambda \theta^{2}}\right)q_{b} & \forall \;X\in \Omega ,\;\forall \;t\geq 0 \end{array}$$

(5c) $$\begin{array}{ccc} \frac{\partial s}{\partial q_{w}} & =-\left(\frac{\tau_{w}}{\lambda \theta_{w}^{2}}\right)q_{w} & \forall \;X\in \Omega ,\;\forall \;t\geq 0 \end{array}$$

(5d) $$\begin{array}{ccc} \frac{\partial s}{\partial {\overset{o}{Q}}_{b}} & =-\left(\frac{\tau_{0,b}\tau_{b}^{2}}{2\lambda \ell^{2}\theta^{2}}\right){\overset{o}{Q}}_{b} & \forall \;X\in \Omega ,\;\forall \;t\geq 0 \end{array}$$

(5e) $$\begin{array}{ccc} \frac{\partial s}{\partial Q_{b}} & =-\left(\frac{3\tau_{1,b}\tau_{b}^{2}}{5\lambda \ell^{2}\theta^{2}}\right)Q_{b} & \forall \;X\in \Omega ,\;\forall \;t\geq 0 \end{array}$$

(5f) $$\begin{array}{ccc} \frac{\partial s}{\partial {\overset{o}{Q}}_{w}} & =-\left(\frac{\tau_{0,w}\tau_{w}^{2}}{2\lambda \ell^{2}\theta_{w}^{2}}\right){\overset{o}{Q}}_{w} & \forall \;X\in \Omega ,\;\forall \;t\geq 0 \end{array}$$

(5g) $$\begin{array}{ccc} \frac{\partial s}{\partial Q_{w}} & =-\left(\frac{3\tau_{1,w}\tau_{w}^{2}}{5\lambda \xi^{2}\ell^{2}\theta_{w}^{2}}\right)Q_{w} & \forall \;X\in \Omega ,\;\forall \;t\geq 0 \end{array}$$

wherein ξ is a non-dimensional parameter that accounts for the possible difference between the values attained by *ℓ* in the bulk and in the Knudsen layer, and $\tau_{b}$, $\tau_{w}$, $\tau_{0,b}$, $\tau_{0,w}$, $\tau_{1,b}$, $\tau_{1,w}$ are the relaxation times of $q_{b}$, $q_{w}$, ${\overset{o}{Q}}_{b}$, ${\overset{o}{Q}}_{w}$, $Q_{b}$, $Q_{w}$, respectively. Before going ahead in our thermodynamic analysis, let us note that Equations (5a)–(5g) are tantamount to assume that in our approach, the specific entropy per unit volume displays the quadratic form

(6) $$\begin{array}{cc} s\left(e,q_{b},q_{w},Q_{b},Q_{w}\right) & =s_{\mathrm{eq}}\left(e\right)-\left(\frac{\tau_{b}}{2\lambda \theta^{2}}\right)q_{b}\cdot q_{b}-\left(\frac{\tau_{0,b}\tau_{b}^{2}}{4\lambda \ell^{2}\theta^{2}}\right)\;{\overset{o}{Q}}_{b}:{\overset{o}{Q}}_{b}-\left(\frac{3\tau_{1,b}\tau_{b}^{2}}{10\lambda \ell^{2}\theta^{2}}\right)Q_{b}^{2}\\ \; & -\left(\frac{\tau_{w}}{2\lambda \theta_{w}^{2}}\right)q_{w}\cdot q_{w}-\left(\frac{\tau_{0,w}\tau_{w}^{2}}{4\lambda \ell^{2}\theta_{w}^{2}}\right)\;{\overset{o}{Q}}_{w}:{\overset{o}{Q}}_{w}-\left(\frac{3\tau_{1,w}\tau_{w}^{2}}{10\lambda \ell^{2}\theta_{w}^{2}}\right)Q_{w}^{2}\; \end{array}$$

∀X∈Ω,∀t>0, wherein $s_{\mathrm{eq}}\left(e\right)$ means the local-equilibrium value of *s*. From Equation (6), it can be seen that in the present approach, *s* clearly displays both a non-equilibrium part related to the bulk thermodynamic fluxes and a non-equilibrium part related to the wall thermodynamic fluxes. A similar assumption has been made in Section 2 of Ref. [22], for example.

Although it has to be recognized that Equation (6) is not the most general form of *s*, we note, however, that it both agrees with the requirement of maximum entropy at equilibrium [4,5,27,28] and with the general theorems of representation of the scalar-valued functions that depend on scalar, vector and tensor variables [29]. It is also worth noticing that Equation (6) encompasses the form of the specific entropy, which is peculiar of EIT (see Equation (9.41a) in Chapter 9 of Ref. [5], for example) whenever the state space is only spanned by the internal energy, the heat flux and the flux of the heat flux (decoupled in its deviatoric and volumetric parts).

By inserting Equations (5a)–(5g) into Equation (4), in fact, from the local balance of *s*,

(7) $$\partial_{t}s+\nabla \cdot J^{\left(s\right)}=\sigma^{\left(s\right)}\;\forall \;X\in \Omega ,\;\forall t>0$$

we are straightforwardly led to the following expression of the specific-entropy production per unit volume $\sigma^{\left(s\right)}$

(8) $$\begin{array}{cc} \sigma^{\left(s\right)}= & -\frac{\nabla \cdot q_{w}}{\theta }-\frac{\tau_{b}}{\lambda \theta^{2}}\left(\partial_{t}q_{b}+\frac{\lambda }{\tau_{b}}\nabla \theta +\nabla \cdot {\overset{o}{Q}}_{b}+\nabla Q_{b}\right)\cdot q_{b}\\ \; & -\frac{\tau_{w}}{\lambda \theta_{w}^{2}}\left(\partial_{t}q_{w}+\nabla {\overset{o}{Q}}_{w}+\nabla Q_{w}\right)\cdot q_{w}\\ \; & -\frac{\tau_{b}^{2}\tau_{0,b}}{2\lambda \ell^{2}\theta^{2}}\left(\partial_{t}{\overset{o}{Q}}_{b}+\frac{2\ell^{2}}{\tau_{b}\tau_{0,b}}{\overset{o}{\nabla q_{b}}}^{\mathrm{sym}}\right)\cdot {\overset{o}{Q}}_{b}\\ \; & -\frac{3\tau_{b}^{2}\tau_{1,b}}{5\lambda \ell^{2}\theta^{2}}\left(\partial_{t}Q_{b}+\frac{5\ell^{2}}{3\tau_{b}\tau_{1,b}}\nabla \cdot q_{b}\right)Q_{b}\\ \; & -\frac{\tau_{w}^{2}\tau_{0,w}}{2\lambda \ell^{2}\theta_{w}^{2}}\left(\partial_{t}{\overset{o}{Q}}_{w}+\frac{2\xi^{2}\ell^{2}}{\tau_{w}\tau_{0,w}}{\overset{o}{\nabla q_{w}}}^{\mathrm{sym}}\right)\cdot {\overset{o}{Q}}_{w}\\ \; & -\frac{3\tau_{w}^{2}\tau_{1,w}}{5\lambda \xi \ell^{2}\theta_{w}^{2}}\left(\partial_{t}Q_{w}+\frac{5\xi^{2}\ell^{2}}{3\tau_{w}\tau_{1,w}}\nabla \cdot q_{w}\right)Q_{w} \end{array}$$

∀X∈Ω and ∀t≥0, if the specific-entropy flux vector is given by the constitutive relation

(9) $$J^{\left(s\right)}=\frac{q_{b}}{\theta }-\frac{\tau_{b}}{\lambda \theta^{2}}\left({\overset{o}{Q}}_{b}+Q_{b}I\right)\cdot q_{b}-\frac{\tau_{w}}{\lambda \theta_{w}^{2}}\left({\overset{o}{Q}}_{w}+Q_{w}I\right)\cdot q_{w}\;\forall \;X\in \Omega ,\;\forall t>0$$

and Equation (3) is employed. For the sake of completeness, we note that in deriving Equation (8), we use the non-equilibrium temperature approximation [5,30] according to which $\frac{1}{\theta^{2}}\approx \frac{1}{\theta_{w}^{2}}\approx \frac{1}{\theta_{\mathrm{eq}}^{2}}$, with $\theta_{\mathrm{eq}}$ being the (constant value of the) local equilibrium temperature; this way, nonlinear terms are easily avoided. For the same reason, we also assumed that all material functions only display constant values. In the nonlinear regime, a more refined model should therefore be pointed out.

Recalling that the second law of thermodynamics states that $\sigma^{\left(s\right)}$ can only attain non-negative values, ∀X∈Ω and ∀t≥0, in our approach, it is sufficient to assume

(10a) $$\begin{array}{ccc} -\frac{q_{b}}{\tau_{b}} & =\partial_{t}q_{b}+\frac{\lambda }{\tau_{b}}\nabla \theta +\nabla \cdot {\overset{o}{Q}}_{b}+\nabla Q_{b} & \forall \;X\in \Omega ,\;\forall \;t\geq 0 \end{array}$$

(10b) $$\begin{array}{ccc} -\frac{q_{w}}{\tau_{w}} & =\partial_{t}q_{w}+\nabla \cdot {\overset{o}{Q}}_{w}+\nabla Q_{w} & \forall \;X\in \Omega ,\;\forall \;t\geq 0 \end{array}$$

(10c) $$\begin{array}{ccc} -\frac{{\overset{o}{Q}}_{b}}{\tau_{0,b}} & =\partial_{t}{\overset{o}{Q}}_{b}+\frac{2\ell^{2}}{\tau_{b}\tau_{0,b}}{\overset{o}{\nabla q_{b}}}^{\mathrm{sym}} & \forall \;X\in \Omega ,\;\forall \;t\geq 0 \end{array}$$

(10d) $$\begin{array}{ccc} -\frac{Q_{b}}{\tau_{1,b}} & =\partial_{t}Q_{b}+\frac{5\ell^{2}}{3\tau_{b}\tau_{1,b}}\nabla \cdot q_{b} & \forall \;X\in \Omega ,\;\forall \;t\geq 0 \end{array}$$

(10e) $$\begin{array}{ccc} -\frac{{\overset{o}{Q}}_{w}}{\tau_{0,w}} & =\partial_{t}{\overset{o}{Q}}_{w}+\frac{2\xi^{2}\ell^{2}}{\tau_{w}\tau_{0,w}}{\overset{o}{\nabla q_{w}}}^{\mathrm{sym}} & \forall \;X\in \Omega ,\;\forall \;t\geq 0 \end{array}$$

(10f) $$\begin{array}{ccc} -\frac{Q_{w}}{\tau_{1,w}} & =\partial_{t}Q_{w}+\frac{5\xi^{2}\ell^{2}}{3\tau_{w}\tau_{1,w}}\nabla \cdot q_{w} & \forall \;X\in \Omega ,\;\forall \;t\geq 0 \end{array}$$

(10g) $$\begin{array}{ccc} \nabla \cdot q_{w} & =0 & \forall \;X\in \Omega ,\;\forall \;t\geq 0 \end{array}$$

in order so that the unilateral constrain arising from Equation (8) always agrees with the second law; in particular, we note that Equations (10a)–(10g) represent the evolution equations of the flux variables. The natural consequence of considerations above is that theoretical model that here we were searching for is the following:

(11a) $$\begin{array}{cc} \partial_{t}e+\nabla \cdot q=0 & \;\forall \;X\in \Omega ,\;\forall \;t\geq 0 \end{array}$$

(11b) $$\begin{array}{cc} \tau_{b}\partial_{t}q_{b}+q_{b}+\lambda \nabla \theta +\tau_{b}\left(\nabla \cdot {\overset{o}{Q}}_{b}+\nabla Q_{b}\right)=0 & \;\forall \;X\in \Omega ,\;\forall \;t\geq 0 \end{array}$$

(11c) $$\begin{array}{cc} \tau_{w}\partial_{t}q_{w}+q_{w}+\tau_{w}\left(\nabla \cdot {\overset{o}{Q}}_{w}+\nabla Q_{w}\right)=0 & \;\forall \;X\in \Omega ,\;\forall \;t\geq 0 \end{array}$$

(11d) $$\begin{array}{cc} \tau_{0,b}\partial_{t}{\overset{o}{Q}}_{b}+{\overset{o}{Q}}_{b}+\frac{2\ell^{2}}{\tau_{b}}{\overset{o}{\nabla q_{b}}}^{\mathrm{sym}}=0 & \;\forall \;X\in \Omega ,\;\forall \;t\geq 0 \end{array}$$

(11e) $$\begin{array}{cc} \tau_{1,b}\partial_{t}Q_{b}+Q_{b}+\frac{5\ell^{2}}{3\tau_{b}}\nabla \cdot q_{b}=0 & \;\forall \;X\in \Omega ,\;\forall \;t\geq 0 \end{array}$$

(11f) $$\begin{array}{cc} \tau_{0,w}\partial_{t}{\overset{o}{Q}}_{w}+{\overset{o}{Q}}_{w}+\frac{2\xi^{2}\ell^{2}}{\tau_{w}}{\overset{o}{\nabla q_{w}}}^{\mathrm{sym}}=0 & \;\forall \;X\in \Omega ,\;\forall \;t\geq 0 \end{array}$$

(11g) $$\begin{array}{cc} \tau_{1,w}\partial_{t}Q_{w}+Q_{w}=0 & \;\forall \;X\in \Omega ,\;\forall \;t\geq 0 \end{array}$$

In closing this section, it seems worth noticing that when the relaxation times of the second-order thermodynamic fluxes are vanishingly small with respect to the relaxation times of the two different heat flux contributions, i.e., whenever $\tau_{0,b},\tau_{1,b}\ll \tau_{b}$ and $\tau_{0,w},\tau_{1,w}\ll \tau_{w}$, Equations (11a)–(11g) directly yield

(12a) $$\begin{array}{cc} \tau_{b}\partial_{t}q_{b}+q_{b}+\lambda \nabla \theta -\ell^{2}\left(\nabla^{2}q_{b}+2\nabla \nabla \cdot q_{b}\right)=0 & \;\forall \;X\in \Omega ,\;\forall \;t\geq 0 \end{array}$$

(12b) $$\begin{array}{cc} \tau_{w}\partial_{t}q_{w}+q_{w}-\xi^{2}\ell^{2}\nabla^{2}q_{w}=0 & \;\forall \;X\in \Omega ,\;\forall \;t\geq 0 \end{array}$$

Whereas Equation (12b) for the wall heat flux is substantially new, Equation (12a) for the bulk heat flux is instead well-known in the literature as the Guyer–Krumhansl [31,32], and it is the usual starting point of the phonon hydrodynamics. The main difference between those two equations is the lack of the temperature gradient in the latter, which can be explained in the following way: the temperature gradient is the driving force (or, alternatively said, the cause) of the propagation of the bulk heat flux, which in turn is then the driving force of the propagation of the wall heat flux. According to this point of view, the relaxation time $\tau_{b}$ could be meant as the time lack between the application of ∇θ and the appearance of $q_{b}$; the relaxation time $\tau_{w}$, instead, could be meant as the time lack between the appearance of $q_{b}$ and that of $q_{w}$.

A similar idea can be found, for example, in Ref. [33] wherein the effect of the inflow boundary conditions on phonon transport in suspended graphene have been studied.

## 3. Longitudinal Heat Transfer in Steady-State Situations

Starting from the theoretical model obtained in Section 2 for the description of the heat transfer at nanoscale, in this section, we deepen the role played by the Knudsen layer (say, the wall heat flux) in possible practical applications of nanosystems. To this end, for example, we note that nanotechnology gave a new life and importance to the study of heat to work conversion. Steady-state devices do this conversion without any macroscopic moving parts through steady-state flows of microscopic particles such as electrons, photons, phonons, i.e., the heat carriers. In steady states, indeed, Equations (11a)–(11g) become

(13a) $$\begin{array}{cc} \nabla \cdot q_{b}=0 & \;\forall \;X\in \Omega ,\;\forall t>0 \end{array}$$

(13b) $$\begin{array}{cc} \nabla \cdot q_{w}=0 & \;\forall \;X\in \Omega ,\;\forall t>0 \end{array}$$

(13c) $$\begin{array}{cc} q_{b}+\lambda \nabla \theta -\ell^{2}\nabla^{2}q_{b}=0 & \;\forall \;X\in \Omega ,\;\forall t>0 \end{array}$$

(13d) $$\begin{array}{cc} q_{w}-\xi^{2}\ell^{2}\nabla^{2}q_{w}=0 & \;\forall \;X\in \Omega ,\;\forall t>0 \end{array}$$

For the mathematical closure of Equations (13a)–(13d), in pursuing our target, we use the following BCs:

(14a) $$\begin{array}{cc} q_{b}\cdot {\hat{t}}_{i}=0 & \;\forall \;X\in \partial \Omega ,\;\forall \;t\geq 0 \end{array}$$

(14b) $$\begin{array}{cc} q_{w}\cdot {\hat{t}}_{i}=C\ell \nabla \left(q_{b}\cdot {\hat{t}}_{i}\right)\cdot \hat{n}-\alpha \ell^{2}\nabla \left[\nabla \left(q_{b}\cdot {\hat{t}}_{i}\right)\cdot \hat{n}\right]\cdot \hat{n} & \;\forall \;X\in \partial \Omega ,\;\forall \;t\geq 0 \end{array}$$

wherein ${\hat{t}}_{i}\left(X\right)$, with i=1,2, are the two unit vectors characterizing the plane, which is tangent to ∂Ω, i.e., ${\hat{t}}_{i}⊥\hat{n}$, ∀X∈∂Ω.

Along with the physical roots, which have been given to $q_{b}$ and $q_{w}$ in Section 1, we observe that Equation (14a) mathematically expresses the idea that the bulk heat flux attains a vanishing value at the boundary. The wall heat flux at the boundary instead has a non-vanishing value, which is given by Equation (14b), wherein *C* and α are two non-dimensional parameters that account for the possible types of reflections (specular and diffusive) of the heat carriers at ∂Ω [16,20,34]. According to the macroscopic approach used in Ref. [21] (see, in particular, therein Section 2), in fact, Equation (14b) states that the heat carriers hitting the system’s boundary are always bounced, and therefore, they contribute to the local value of the heat flux. This contribution, which gives the actual value of $q_{w}$ at ∂Ω in the present approach, is only related to the spatial derivatives of $q_{b}$ because the hitting heat carriers come from the bulk. Along with Ref. [21], here, we estimate the two parameters *C* and α as

(15a) $$\begin{array}{cc} \; & C=\frac{2}{3}\left(\frac{3-\nu p^{3}}{\nu }-\frac{3}{2}\cdot \frac{1-p^{2}}{\mathrm{Kn}}\right), \end{array}$$

(15b) $$\begin{array}{cc} \; & \alpha =\frac{1}{4}\left[p^{4}+\frac{2}{{\mathrm{Kn}}^{2}}\left(1-p^{2}\right)\right] \end{array}$$

wherein Kn is the so-called Knudsen number, i.e., the ratio between the heat-carrier mean-free path and the characteristic length of the system at hand, $p=\min\{{\mathrm{Kn}}^{-1};1\}$. In Equations (15a) and (15b), moreover, ν∈(0,1) is the momentum accommodation coefficient: it turns out information about the portion of the total wall-colliding heat carriers are diffusively reflected back by the boundary ∂Ω. In particular, the larger the ν, the larger the number of the heat carriers, which are backwardly bounced. The (non-dimensional) parameter ν is clearly related to the roughness of the boundary, which can be characterized by the root-mean square value of the roughness fluctuations and the average distance between the roughness’ peaks [35]. Small values of ν should therefore be used in the case of a smooth boundary, whereas large values of ν should be employed in the case of a rough boundary.

From the practical point of view, in applying Equation (14b), here, we only note that the term $\nabla \left(q_{b}\cdot {\hat{t}}_{i}\right)\cdot \hat{n}$ has to be evaluated in such a way that it is a non-negative term; more comments about the influence of Equations (14a) and (14b) on the solutions of Equations (13a)–(13d) will be given in Section 4.

### 3.1. The Case of a Two-Dimensional Nanolayer

**Figure 1:**
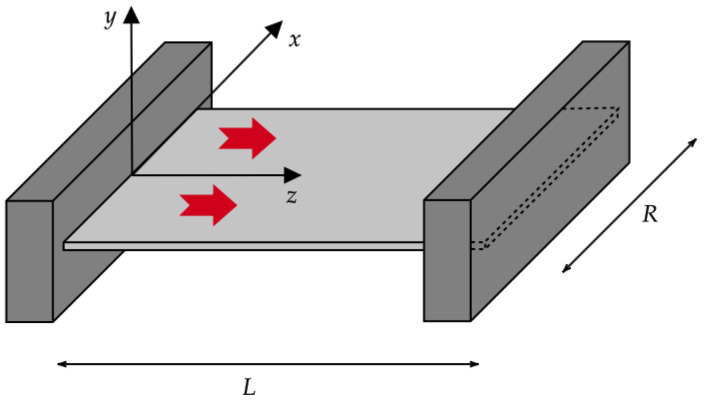
Sketch of a 2D nanolayer connected with two different operating nanodevices, namely, the left-hand and right-hand parallelepipeds. The height of the nanolayer (i.e., the characteristic size along the *y* axis in the figure), being very small, can be neglected with respect to its length *L* and width *R* (that is, the characteristic sizes along the *z* and *x* axes in the figure, respectively). This means that *x* and *z* are the only Cartesian coordinates that matter. The generic cross section xy, moreover, can be reduced to a simple line along the *x* axis. The large (red in figure) arrows stand for the local heat flux vector: it only propagates along the *z* axis from the (hot) left-hand side to the (cold) right-hand side. From the figure, it can be also seen that $x\in \left[-\frac{R}{2},\frac{R}{2}\right]$ and $z\in \left[0,L\right]$, with R<L.

Here, we focus on a two-dimensional (2D) nanolayer longitudinally crossed by a heat flux, i.e., we assume that $q_{b}\left(X\right)=q_{b}\left(x,z\right)\hat{z}$ and $q_{w}\left(X\right)=q_{w}\left(x,z\right)\hat{z}$, with $\hat{z}$ being the longitudinal unit vector of the *z*-axis, as well as θ=θ(x,z). This could be the case, for example, when two different operating nanodevices are connected via a nanolayer (see Figure 1 for a qualitative sketch). In this situation, Equations (13a)–(13d) become

(16a) $$\begin{array}{cc} \frac{\partial q_{b}}{\partial z}=0 & \;\forall \;\left(x,z\right)\in \left[-\frac{R}{2},\frac{R}{2}\right]\times \left[0,L\right] \end{array}$$

(16b) $$\begin{array}{cc} \frac{\partial q_{w}}{\partial z}=0 & \;\forall \;\left(x,z\right)\in \left[-\frac{R}{2},\frac{R}{2}\right]\times \left[0,L\right] \end{array}$$

(16c) $$\begin{array}{cc} \frac{\partial \theta }{\partial x}=0 & \;\forall \;\left(x,z\right)\in \left[-\frac{R}{2},\frac{R}{2}\right]\times \left[0,L\right] \end{array}$$

(16d) $$\begin{array}{cc} q_{b}+\lambda \frac{\partial \theta }{\partial z}-\ell^{2}\left(\frac{\partial^{2}q_{b}}{\partial x^{2}}+\frac{\partial^{2}q_{b}}{\partial z^{2}}\right)=0 & \;\forall \;\left(x,z\right)\in \left[-\frac{R}{2},\frac{R}{2}\right]\times \left[0,L\right] \end{array}$$

(16e) $$\begin{array}{cc} q_{w}-\xi^{2}\ell^{2}\left(\frac{\partial^{2}q_{w}}{\partial x^{2}}+\frac{\partial^{2}q_{w}}{\partial z^{2}}\right)=0 & \;\forall \;\left(x,z\right)\in \left[-\frac{R}{2},\frac{R}{2}\right]\times \left[0,L\right] \end{array}$$

From Equations (16a) and (16b), it is an easy matter to infer that the longitudinal heat flux is independent of the transversal section, namely, $q_{b}=q_{b}\left(x\right)$ and $q_{w}=q_{w}\left(x\right)$, for the special case of the system at hand in steady states. These considerations allow us to rewrite the BCs in Equations (14a) and (14b) as

(17a) $$\begin{array}{cc} \; & q_{b}\left(\frac{R}{2}\right)=0 \end{array}$$

(17b) $$\begin{array}{cc} \; & q_{b}\left(-\frac{R}{2}\right)=0 \end{array}$$

(17c) $$\begin{array}{cc} \; & q_{w}\left(\frac{R}{2}\right)=-C\ell {\left.\frac{dq_{b}}{dx}\right|}_{x=R/2}-\alpha \ell^{2}{\left.\frac{d^{2}q_{b}}{dx^{2}}\right|}_{x=R/2} \end{array}$$

(17d) $$\begin{array}{cc} \; & q_{w}\left(-\frac{R}{2}\right)=C\ell {\left.\frac{dq_{b}}{dx}\right|}_{x=-R/2}-\alpha \ell^{2}{\left.\frac{d^{2}q_{b}}{dx^{2}}\right|}_{x=-R/2} \end{array}$$

The temperature, instead, is independent of the longitudinal section, namely, we have

(18) θ=θ(z)

as it directly arises from Equation (16c). Since neither $q_{b}$ nor $q_{w}$ varies along *z*, from Equation (18), it directly follows that the temperature field θ can only linearly vary with *z*, namely, in the present case, we have $\frac{\partial \theta }{\partial z}=c$, with $c\in R^{-}$.

For computational needs, we now introduce the following non-dimensional variables

(19) $$x=\frac{x}{R},\;z=\frac{z}{L}$$

and the following non-dimensional quantities

(20) $$T=\frac{\theta -\theta_{0}}{\theta_{0}},\;h_{b}=\left(\frac{L_{\mathrm{ref}}}{\lambda \theta_{0}}\right)q_{b},\;h_{w}=\left(\frac{L_{\mathrm{ref}}}{\lambda \theta_{0}}\right)q_{w}$$

with $\theta_{0}$ and $L_{\mathrm{ref}}$, respectively, standing for a suitable reference temperature and a suitable reference length. Since in this case the heat flux only flows along the $\hat{z}$ direction, it seems logical to assume $L_{\mathrm{ref}}=L$.

#### 3.1.1. The Behavior of the Heat Flux Vectors

By means of Equations (16)–(20), straightforward calculations allow us to obtain the following behaviors of the unknown (non-dimensional) basic fields $h_{b}$ and $h_{w}$ in each transversal section $z^{★}\in \left[0,1\right]$ of the nanolayer:

(21a) $$\begin{array}{ccc} h_{b}\left(x\right) & =\Delta T^{•}\left[1-\frac{\cosh\left(\frac{x}{\mathrm{Kn}}\right)}{\cosh\left(\frac{1}{2\mathrm{Kn}}\right)}\right] & \forall \;x\in \left[-\frac{1}{2},\frac{1}{2}\right] \end{array}$$

(21b) $$\begin{array}{ccc} h_{w}\left(x\right) & =\Delta T^{•}\left[C\tanh\left(\frac{1}{2\mathrm{Kn}}\right)-\alpha \right]\frac{\cosh\left(\frac{x}{\xi \mathrm{Kn}}\right)}{\cosh\left(\frac{1}{2\xi \mathrm{Kn}}\right)} & \forall \;x\in \left[-\frac{1}{2},\frac{1}{2}\right] \end{array}$$

wherein Kn=ℓ/R is the Knudsen number and $\Delta T^{•}\in R^{+}$ turns out information about the temperature gradient between z=0 and z=1.

According to the constitutive assumption in Equation (1), the following behavior of the (non-dimensional) heat flux field is therefore recovered in the present approach in the case of a 2D nanolayer:

(22) $$h\left(x\right)=\Delta T^{•}\left\{1-\frac{\cosh\left(\frac{x}{\mathrm{Kn}}\right)}{\cosh\left(\frac{1}{2\mathrm{Kn}}\right)}+\left[C\tanh\left(\frac{1}{2\mathrm{Kn}}\right)-\alpha \right]\frac{\cosh\left(\frac{x}{\xi \mathrm{Kn}}\right)}{\cosh\left(\frac{1}{2\xi \mathrm{Kn}}\right)}\right\}$$

**Figure 2:**
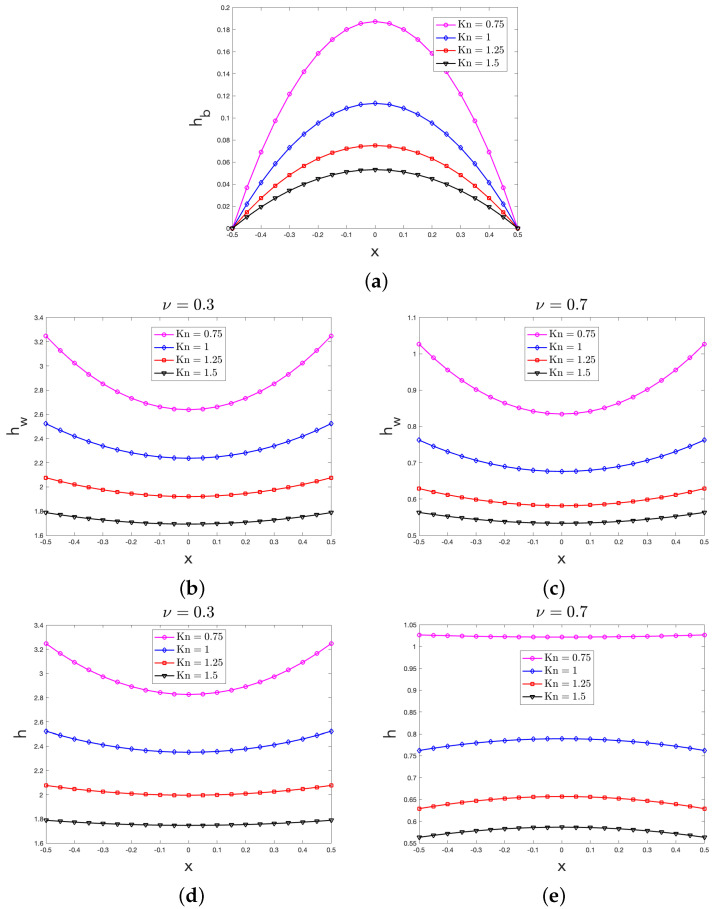
Behavior of the: non-dimensional bulk heat flux $h_{b}$ arising from Equation (21a) (**a**); non-dimensional wall heat flux $h_{w}$ arising from Equation (21b) both when ν=0.3 (**b**) and when ν=0.7 (**c**); non-dimensional heat flux h arising from Equation (22) both when ν=0.3 (**d**) and when ν=0.7 (**e**). According to Equation (19), in the figure, the boundaries are set at $x=-\frac{1}{2}$ and $x=\frac{1}{2}$.

By means of Equations (21) and (22), in terms of the non-dimensional variable x spanning along the transversal section of the nanolayer, in Figure 2, we plot the behaviors of $h_{b}$, $h_{w}$ and h for different values of Kn both when ν=0.3 (smooth boundary) and when ν=0.7 (rough boundary); for the sake of computation, in obtaining those behaviors, we assumed $\Delta T^{•}=1$ and ξ=1.

#### 3.1.2. The Effective Thermal Conductivity

By using the (non-dimensional) heat flux behavior in Equation (22), we may estimate that in the generic transversal section of the nanolayer, the thermal conductivity displays the following (non-dimensional) effective value:

(23) $$\begin{array}{cc} \lambda_{\mathrm{eff}}=\frac{H_{\mathrm{tot}}}{\Delta T^{•}} & =\frac{\int_{-1/2}^{1/2}h\left(x\right)dx}{\Delta T^{•}}\\ \; & =1-2\mathrm{Kn}\tanh\left(\frac{1}{2\mathrm{Kn}}\right)\left[1-\xi C\tanh\left(\frac{1}{2\xi \mathrm{Kn}}\right)\right]-2\xi \alpha \tanh\left(\frac{1}{2\xi \mathrm{Kn}}\right) \end{array}$$

**Figure 3:**
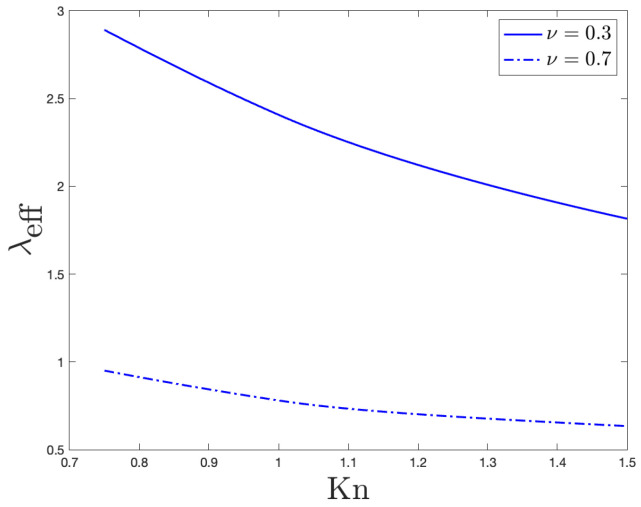
Behavior of the non-dimensional effective thermal conductivity versus the Knudsen number. That behavior arises from Equation (23), when ν=0.3 (solid line) and when ν=0.7 (dashdotted line).

In Figure 3, we plot the behavior of the non-dimensional effective thermal conductivity ($\lambda_{\mathrm{eff}}$) versus the Knudsen number (Kn), both when the momentum accommodation coefficient ν=0.3 (smooth boundary) and when ν=0.7 (rough boundary). In evaluating Equation (23), we clearly still assumed $\Delta T^{•}=1$ and ξ=1.

### 3.2. The Case of a Nanowire

**Figure 4:**
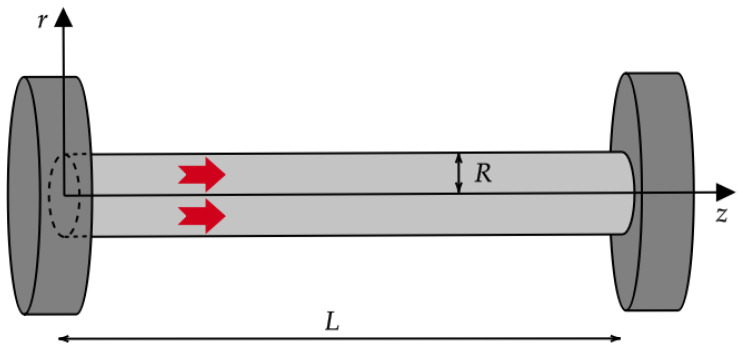
Sketch of a nanowire connected with two different operating nanodevices, namely, the left-hand and right-hand cylinders. The radius *R* of the nanowire (i.e., the characteristic size along the *r* axis) is generally smaller than its length *L* (that is, the characteristic size along the *z* axis). When the nanowire is homogeneous, *r* and *z* are the only cylindrical coordinates that matter. The large (red in figure) arrows stand for the local heat flux vector: it only propagates along the *z* axis from the (hot) left-hand side to the (cold) right-hand side. From the figure, it can also be seen that r∈[0,R] and z∈[0,L], with R<L.

Here, we focus on a nanowire crossed by a longitudinal heat flux, i.e., we assume that $q_{b}\left(X\right)=q_{b}\left(r,z\right)\hat{z}$ and $q_{w}\left(X\right)=q_{w}\left(r,z\right)\hat{z}$, with $\hat{z}$ being the longitudinal unit vector of the *z* axis, as well as θ=θ(x,z) again. This could be the case, for example, when two different operating nanodevices are connected via a nanowire (see Figure 4 for a qualitative sketch). In this situation, Equations (13a)–(13d) become

(24a) $$\begin{array}{cc} \frac{\partial q_{b}}{\partial z}=0 & \;\forall \;(r,z)\in [0,R]\times [0,L] \end{array}$$

(24b) $$\begin{array}{cc} \frac{\partial q_{w}}{\partial z}=0 & \;\forall \;(r,z)\in [0,R]\times [0,L] \end{array}$$

(24c) $$\begin{array}{cc} \frac{\partial \theta }{\partial r}=0 & \;\forall \;(r,z)\in [0,R]\times [0,L] \end{array}$$

(24d) $$\begin{array}{cc} q_{b}+\lambda \frac{\partial \theta }{\partial z}-\ell^{2}\left[\frac{1}{r}\frac{\partial }{\partial r}\left(r\frac{\partial q_{b}}{\partial r}\right)+\frac{\partial^{2}q_{b}}{\partial z^{2}}\right]=0 & \;\forall \;(r,z)\in [0,R]\times [0,L] \end{array}$$

(24e) $$\begin{array}{cc} q_{w}-\xi^{2}\ell^{2}\left[\frac{1}{r}\frac{\partial }{\partial r}\left(r\frac{\partial q_{w}}{\partial r}\right)+\frac{\partial^{2}q_{w}}{\partial z^{2}}\right]=0 & \;\forall \;(r,z)\in [0,R]\times [0,L] \end{array}$$

From Equations (24a) and (24b), it is an easy matter to infer that the longitudinal heat flux is independent of the transversal section, namely, $q_{b}=q_{b}\left(r\right)$ and $q_{w}=q_{w}\left(r\right)$, for the special case of the system at hand; as a consequence, the BCs in Equations (14a) and (14b) read

(25a) $$\begin{array}{cc} \; & q_{b}\left(R\right)=0 \end{array}$$

(25b) $$\begin{array}{cc} \; & q_{w}\left(R\right)=-C\ell {\left.\frac{dq_{b}}{dr}\right|}_{r=R}-\alpha \ell^{2}{\left.\frac{d^{2}q_{b}}{dr^{2}}\right|}_{r=R} \end{array}$$

From Equation (24c), one is again led to Equation (18): also in this case, the temperature is independent of the longitudinal section.

For computational needs, here, we use the following non-dimensional variables

(26) $$r=\frac{r}{R},\;z=\frac{z}{L}$$

whereas the non-dimensional heat fluxes and temperature are still defined by Equation (20).

#### 3.2.1. The Behavior of the Heat Flux Vector

From the non-dimensional version of Equations (24a)–(24e), straightforward calculations allow us to then obtain the following behaviors of the unknown (non-dimensional) basic fields $h_{b}$ and $h_{w}$ in each transversal section $z^{★}\in \left[0,1\right]$ of the nanowire:

(27a) $$\begin{array}{ccc} h_{b}\left(r\right) & =\Delta T^{•}\left[1-\frac{I_{0}\left(\frac{r}{\mathrm{Kn}}\right)}{I_{0}\left(\frac{1}{\mathrm{Kn}}\right)}\right] & \forall \;r\in [0,1] \end{array}$$

(27b) $$\begin{array}{ccc} h_{w}\left(r\right) & =\Delta T^{•}\left[\left(C-\alpha \mathrm{Kn}\right)\frac{I_{1}\left(\frac{1}{\mathrm{Kn}}\right)}{I_{0}\left(\frac{1}{\mathrm{Kn}}\right)}+\alpha \right]\frac{I_{0}\left(\frac{r}{\xi \mathrm{Kn}}\right)}{I_{0}\left(\frac{1}{\xi \mathrm{Kn}}\right)} & \forall \;r\in [0,1] \end{array}$$

For the sake of clarity, we note that in Equations (27a) and (27b), $I_{\gamma }\left(\cdot \right)$ is the γ-order modified Bessel function of the first kind of the indicated argument, with γ∈{0;1}. Therein, Kn and $\Delta T^{•}$, moreover, have the same physical meanings of Section 3.1.1. According to the constitutive assumption in Equation (1), the following behavior of the (non-dimensional) heat flux field is then recovered in the present approach in the case of a nanowire:

(28) $$h\left(r\right)=\Delta T^{•}\left\{1-\frac{I_{0}\left(\frac{r}{\mathrm{Kn}}\right)}{I_{0}\left(\frac{1}{\mathrm{Kn}}\right)}+\left[\left(C-\alpha \mathrm{Kn}\right)\frac{I_{1}\left(\frac{1}{\mathrm{Kn}}\right)}{I_{0}\left(\frac{1}{\mathrm{Kn}}\right)}+\alpha \right]\frac{I_{0}\left(\frac{r}{\xi \mathrm{Kn}}\right)}{I_{0}\left(\frac{1}{\xi \mathrm{Kn}}\right)}\right\}\;\forall \;r\in \left[0,1\right]$$

**Figure 5:**
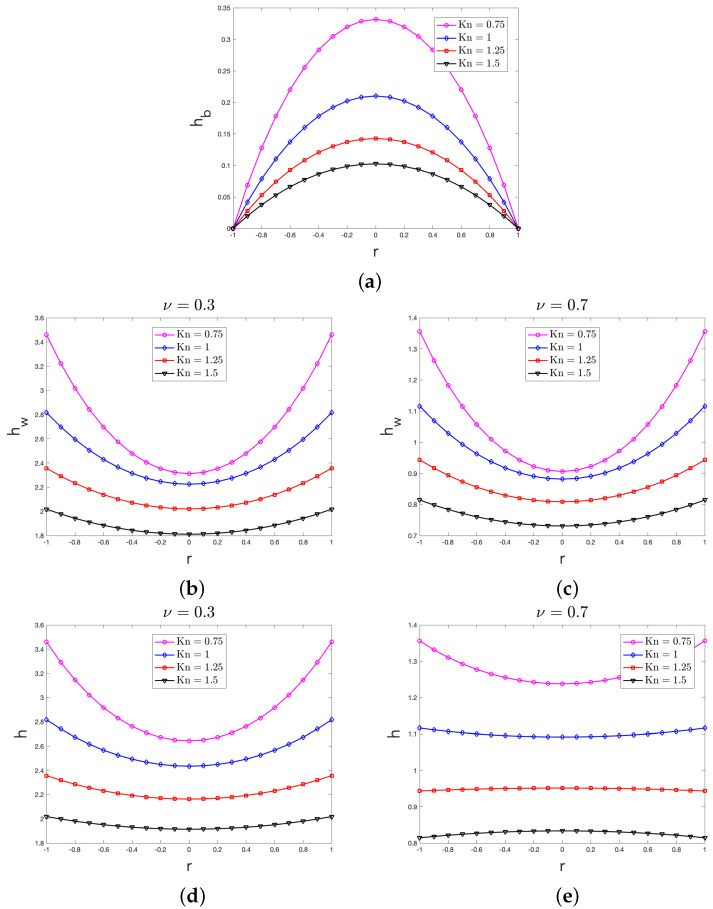
Behavior of the: non-dimensional bulk heat flux $h_{b}$ arising from Equation (27a) (**a**); non-dimensional wall heat flux $h_{w}$ arising from Equation (27b), both when ν=0.3 (**b**) and when ν=0.7 (**c**); non-dimensional heat flux h arising from Equation (28), both when ν=0.3 (**d**) and when ν=0.7 (**e**). According to Equation (26), in the figure, the boundaries are set at r=−1 and r=1.

By means of Equations (27a), (27b) and (28), in terms of the non-dimensional variable r (indicating the radial distance from the center of the transversal section of the nanowire), in Figure 5, we plot the behaviors of $h_{b}$, $h_{w}$ and h for different values of Kn both when ν=0.3 (smooth boundary) and when ν=0.7 (rough boundary); for the sake of computation, in obtaining those behaviors, we assumed $\Delta T^{•}=1$ and ξ=1.

#### 3.2.2. The Effective Thermal Conductivity

By using the (non-dimensional) heat flux behavior in Equation (28), we may estimate that in the generic transversal section $z^{★}$ of the nanowire, the thermal conductivity displays the following (non-dimensional) effective value:

(29) $$\begin{array}{cc} \lambda_{\mathrm{eff}}=\frac{H_{\mathrm{tot}}}{\pi \Delta T^{•}} & =\frac{\int_{0}^{1}2\pi h\left(r\right)rdr}{\pi \Delta T^{•}}\\ \; & =1-2\mathrm{Kn}\left\{\left[\frac{I_{0}\left(\frac{1}{\xi \mathrm{Kn}}\right)}{I_{1}\left(\frac{1}{\xi \mathrm{Kn}}\right)}-\left(C-\alpha \mathrm{Kn}\right)\xi \right]\frac{I_{1}\left(\frac{1}{\mathrm{Kn}}\right)}{I_{0}\left(\frac{1}{\mathrm{Kn}}\right)}-\alpha \xi \right\}\frac{I_{1}\left(\frac{1}{\xi \mathrm{Kn}}\right)}{I_{0}\left(\frac{1}{\xi \mathrm{Kn}}\right)} \end{array}$$

**Figure 6:**
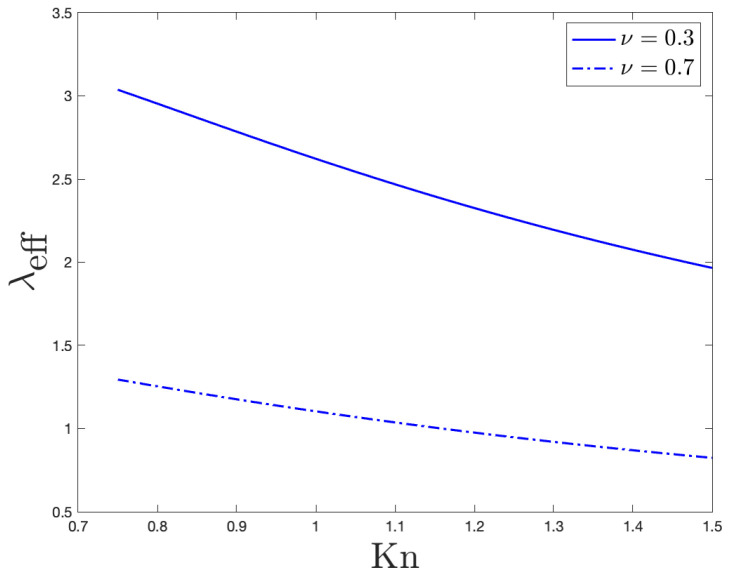
Behavior of the non-dimensional effective thermal conductivity versus the Knudsen number. That behavior arises from Equation (29), when ν=0.3 (solid line) and when ν=0.7 (dashdotted line). For the sake of computation, in obtaining the plotted results, we assumed $\Delta T^{•}=1$ and ξ=1.

In Figure 6, we plot the behavior of the non-dimensional effective thermal conductivity ($\lambda_{\mathrm{eff}}$) versus the Knudsen number (Kn), both when the momentum accommodation coefficient ν=0.3 (smooth boundary) and when ν=0.7 (rough boundary). In evaluating Equation (29), we assumed $\Delta T^{•}=1$ and ξ=1.

## 4. Summary and Conclusions

Aiming at the goal of improving the theoretical models for the description of thermal transport at nanoscale, in the present paper, we especially investigate how the phonon–boundary interactions may influence the actual value of the heat flux in a rigid body Ω. Since those interactions should be the main scattering mechanism of the heat carriers near the boundary ∂Ω, we have introduced here the concept of the Knudsen layer, that is, a strip close to ∂Ω, the characteristic dimension of which is of the order of the mean-free path of the heat carriers. The presence of the Knudsen layer in particular allowed us to account for two different contributions to the local heat flux q: the bulk heat flux $q_{b}$ and the wall heat flux $q_{w}$. Both those heat flux vectors have their own evolution equations, which are in agreement with the second law of thermodynamics, as it has been discussed in Section 2. In order to scrutinize the way $q_{b}$ and $q_{w}$ behave, as well as that they may influence q in steady states, in Section 3, we have put our attention both on nanolayers and on nanowires. Below, we spend some useful comments about the main results therein obtained.

### 4.1. Comments on the Bulk Heat Flux Profile

For all the values of the Knudsen considered in Section 3, Figure 2a (for the case of a nanolayer) and Figure 5a (for the case of a nanowire) show that the bulk heat flux contribution to the local heat flux displays a non-uniform profile. Since in our approach in each point of the system the value of the heat flux is only related to the particular type of phonon scattering [20], the above concave profile was indeed expected because the role of the boundary scattering in the bulk of the system is practically neglected with respect to the other scattering mechanisms, as it is also stated by the BCs in Equation (14a). In accordance with this point of view, one can indeed alternatively claim that in the bulk, the diffuse boundary scattering is the main mechanism for momentum loss of the phonons, thus justifying that $h_{b}$ is vanishingly small near the system’s boundary.

By looking at those two figures, moreover, it can also be seen that for increasing values of Kn, the non-uniform profile of $h_{b}$ becomes less marked: in fact, it tends to a uniform profile for large enough values of Kn. High values of the Knudsen number, indeed, characterize the so-called ballistic regime of heat transfer, wherein the phonons in the bulk only undergo to scant scattering’s mechanisms [34], thus leading to an almost flat profile.

### 4.2. Comments on the Wall Heat Flux Profile

For all the values of the Knudsen considered in Section 3, Figure 2b,c (for the case of a nanolayer) and Figure 5b,c (for the case of a nanowire) show that the wall heat flux contribution to the local heat flux also displays a non-uniform profile. Unlike what was recovered for $h_{b}$, the profile of $h_{w}$ is instead convex. This behavior seems rather natural and indeed expected in our approach, wherein (we recall that) the value of the wall heat flux in each point X∈Ω is only related to the number of the heat carriers, which are redirected afterwards with the different scattering mechanisms happening in X [20]. In the Knudsen layer, wherein the boundary scattering prevails over the other scattering mechanisms, the frequency of interactions between the heat carriers and the lateral walls is large in the zones close to ∂Ω; far from ∂Ω, instead, the boundary scattering starts losing its importance. As a consequence, the larger the distance from the domain’s boundary, the smaller the value of $h_{w}$, thus explaining the reason why the wall heat flux is characterized by a convex profile with a minimum value in the center of the transversal section.

Since in our approach the actual value of the wall heat flux is strictly related to the particular type of reflections of the phonons at the boundary, as indeed mathematically stated by the BCs in Equation (14b), it is also logical that the smaller the accommodation coefficient ν (i.e., the smoother the system’s boundary), the larger the actual value of $h_{w}$. The differences between the actual values of $h_{w}$ in Figure 2b,c for the case of a nanolayer and between the actual values of $h_{w}$ in Figure 5b,c for the case of a nanowire were therefore also expected.

From those four figures, it can be finally observed that for increasing values of Kn, the non-uniform profile displayed by $h_{w}$ becomes less marked, tending towards a uniform profile for large enough values of Kn, as it is expected in the ballistic regime [34].

### 4.3. Comments on the Heat Flux Profile

For all the values of the Knudsen considered in Section 3, Figure 2d,e (for the case of a nanolayer) and Figure 5d,e (for the case of a nanowire) clearly point out the importance of the Knudsen layer, wherein the phonon–boundary scattering is mainly accounted; ∀X∈Ω, in fact, the local heat flux values are always larger than the corresponding values of the bulk heat flux, which have been instead obtained by neglecting the contribution of the wall reflections of phonons. Those figures, moreover, show that the local heat flux displays a non-uniform profile that may be either concave or convex, depending on the particular situation. These results seem to deserve a deeper investigation in future analyses. Depending on the particular situation, in fact, it could be recovered either that the larger the distance from the boundary, the larger the local heat flux values, or the opposite case. At the present stage, we may only observe that those results clearly point out that the phonon–wall scattering should be treated with care and rightly accounted if one aims at a better thermal management of nanosystems.

It seems worth noticing that those figures also show that when the Knudsen number declines, the maximum value of the local heat flux increases: this feature of predicted results, which is also peculiar of $h_{b}$ and $h_{w}$, has been also recovered in Ref. [33]. From the physical point of view, a possible explanation of this trend may be the following: when Kn increases, the Knudsen layer enlarges in such a way that it starts to pervade the main part of Ω. Since, therein, the frequency of phonon–boundary interactions is high, then the phonon–boundary scattering become the main event in Ω. As a consequence, the reduction in the local heat flux amplitude due to the momentum loss becomes homogeneous in the whole system, thus leading to a heat flux profile that is practically flat, as it is shown in Figure 2d,e (for the case of a nanolayer) and Figure 5d,e (for the case of a nanowire) when Kn=1.5, for example.

For any given value of Kn, Equation (22) (for the case of a nanolayer) and Equation (28) (for the case of a nanowire) finally predict that the value of h increases for a decreasing value of ν, that is, the smoother the system’s boundary, the larger the value of the local heat flux. This feature was indeed naturally expected in our hydrodynamical approach to the heat transfer at nanoscale.

### 4.4. Comments on the Effective Thermal Conductivity

For increasing values of the Knudsen number, Figure 3 (for the case of a nanolayer) and Figure 6 (for the case of a nanowire) point out that the effective thermal conductivity reduces. Those trends comply with experimental observations: significant reductions in thermal conductivity have been, in fact, found in different nanowires (especially made of silicon) with some of them beyond what Fourier-based theories can explain [36]. Such a reduction in our approach displays a linear-like dependence on the characteristic dimension of the system, which, here, is the length of the transversal section in the case of nanolayers and the radius of the transversal section in the case of nanowires.These linear behaviors agree with the theoretical results obtained in Ref. [34] for the effective thermal conductivity. They indeed also comply with experimental observations in nanowires; to this end, see Refs. [37,38,39], for example.

It could, finally, be interesting to draw the attention of the readers on the different values of $\lambda_{\mathrm{eff}}$ that are predicted by our theoretical model: depending on the roughness’ degree of the boundary (i.e., depending on the values of ν), in fact, substantially different values of the effective thermal conductivity can be observed in Figure 3 and Figure 6. In more details, the present analysis points out that the smaller the ν, the larger the $\lambda_{\mathrm{eff}}$. These results could be important at nanoscale since a better device’s cooling is crucial for the correct use of nanodevices.

### 4.5. Final Remark

Except for the case of Kn=0.75, in the present paper, we only assumed *large enough* values of the Knudsen number in such a way that the Knudsen layer fully pervades the bulk of Ω.

An intriguing question may be, however, the following: how to deal with the Knudsen layer whenever Kn attains *small enough* values, as, for example, whenever Kn is of the order $10^{-1}$? Since in these situations the Knudsen layer practically reduces to the systems’ boundary ∂Ω, we feel that the approach used in Refs. [20,21] should be preferred with respect to the approach used in the present paper whenever Kn is small enough. 

## Data Availability

Data is contained within the article.

## Nomenclature

List of the Physical Quantities Involved in This Paper

**Symbol**	**Meaning**	**Unit of Measurement**
θ	non-equilibrium temperature	K
$\theta_{w}$	value of θ at the wall	K
$\theta_{\mathrm{eq}}$	local-equilibrium temperature	K
$\theta_{0}$	reference temperature	K
*e*	specific internal energy	$\frac{J}{m^{3}}$
q	local heat flux	$\frac{W}{m^{2}}$
$q_{b}$	bulk heat flux	$\frac{W}{m^{2}}$
$Q_{b}$	flux of $q_{b}$	$\frac{W}{ms}$
${\overset{o}{Q}}_{b}$	deviatoric (traceless) part of $Q_{b}$	$\frac{W}{ms}$
$Q_{b}$	volumetric part of $Q_{b}$	$\frac{W}{ms}$
$q_{w}$	wall heat flux	$\frac{W}{m^{2}}$
$Q_{w}$	flux of $q_{w}$	$\frac{W}{ms}$
${\overset{o}{Q}}_{w}$	deviatoric (traceless) part of $Q_{w}$	$\frac{W}{ms}$
$Q_{b}$	volumetric part of $Q_{b}$	$\frac{W}{ms}$
$\tau_{b}$	relaxation time of $q_{b}$	s
$\tau_{w}$	relaxation time of $q_{w}$	s
$\tau_{0,b}$	relaxation time of ${\overset{o}{Q}}_{b}$	s
$\tau_{0,w}$	relaxation time of ${\overset{o}{Q}}_{w}$	s
$\tau_{1,b}$	relaxation time of $Q_{b}$	s
$\tau_{1,w}$	relaxation time of $Q_{w}$	s
λ	thermal conductivity	$\frac{W}{m K}$
*ℓ*	mean-free path of phonons	m
*s*	specific entropy per unit volume	$\frac{J}{m^{3}K}$
$J^{\left(s\right)}$	specific-entropy flux per unit volume	$\frac{W}{m^{2}K}$
$\sigma^{\left(s\right)}$	specific-entropy production per unit volume	$\frac{W}{m^{3}K}$
T	non-equilibrium temperature	
$\Delta T^{•}$	temperature gradient	
$h_{b}$	bulk heat flux	
$h_{w}$	wall heat flux	
h	$h_{b}+h_{w}$	
$H_{\mathrm{tot}}$	total heat flux	
Kn	Knudsen number	
$\left(C,\alpha \right)$	parameters accounting for the reflections of the	
	heat carriers at the walls	
*p*	$\min\{{\mathrm{Kn}}^{-1};1\}$	
ν	momentum accommodation coefficient	
ξ	parameter accounting for the possible difference	
	of *ℓ* in the bulk and in the Knudsen layer	
$\lambda_{\mathrm{eff}}$	effective thermal conductivity	

## References

[1] Chen G. (2005). Nanoscale Energy Transport and Conversion—A Parallel Treatment of Electrons, Molecules, Phonons, and Photons.

[2] Ván P. (2001). Weakly nonlocal irreversible thermodynamics—The Guyer-Krumhansl and the Cahn-Hilliard equations. Phys. Lett. A.

[3] Cao B.-Y., Guo Z.-Y. (2007). Equation of motion of a phonon gas and non-Fourier heat conduction. J. Appl. Phys..

[4] Lebon G., Jou D., Casas-Vázquez J. (2008). Understanding Non-Equilibrium Thermodynamics.

[5] Jou D., Casas-Vázquez J., Lebon G. (2010). Extended Irreversible Thermodynamics, 4th revised ed..

[6] Tzou D.Y. (2014). Macro- to Microscale Heat Transfer: The Lagging Behaviour.

[7] Cahill D.G., Braun P.V., Chen G., Clarke D.R., Fan S., Goodson K.E., Keblinski P., King W.P., Mahan G.D., Majumdar A. (2014). Nanoscale thermal transport. II. 2003–2012. Appl. Phys. Rev..

[8] Dong Y. (2016). Dynamical Analysis of Non-Fourier Heat Conduction and Its Application in Nanosystems.

[9] Sellitto A., Cimmelli V.A., Jou D. (2016). Linear and Nonlinear Heat-Transport Equations. Mesoscopic Theories of Heat Transport in Nanosystems.

[10] Kovács R., Madjarević D., Simić S., Ván P. (2021). Non-equilibrium theories of rarefied gases: Internal variables and extended thermodynamics. Continuum Mech. Thermodyn..

[11] Fugallo G., Lazzeri M., Paulatto L., Mauri F. (2013). Ab initio variational approach for evaluating lattice thermal conductivity. Phys. Rev. B.

[12] Li W., Carrete J., Katcho N.A., Mingo N. (2014). ShengBTE: A solver of the Boltzmann transport equation for phonons. Comput. Phys. Commun..

[13] Zou J.-H., Cao B.-Y. (2017). Phonon thermal properties of graphene on h-BN from molecular dynamics simulations. J. Appl. Phys..

[14] He J., Li D., Ying Y., Feng C., He J., Zhong C., Zhou H., Zhou P., Zhang G. (2019). Orbitally driven giant thermal conductance associated with abnormal strain dependence in hydrogenated graphene-like borophene. npj Comput. Mater..

[15] Guo Y., Wang M. (2015). Phonon hydrodynamics and its applications in nanoscale heat transport. Phys. Rep..

[16] Sellitto A., Cimmelli V.A., Jou D. (2016). Mesoscopic Description of Boundary Effects and Effective Thermal Conductivity in Nanosystems: Phonon Hydrodynamics. Mesoscopic Theories of Heat Transport in Nanosystems.

[17] Guo Y., Wang M. (2018). Phonon hydrodynamics for nanoscale heat transport at ordinary temperatures. Phys. Rev. B.

[18] Beardo A., Hennessy M.G., Sendra L., Camacho J., Myers T.G., Bafaluy J., Alvarez F.X. (2020). Phonon hydrodynamics in frequency-domain thermoreflectance experiments. Phys. Rev. B.

[19] Lee S., Broido D., Esfarjani K., Chen G. (2015). Hydrodynamic phonon transport in suspended graphene. Nat. Comm..

[20] Bochicchio I., Giannetti F., Sellitto A. (2022). Heat transfer at nanoscale and boundary conditions. Z. Angew. Math. Phys..

[21] Bochicchio I., Sellitto A., Munafó C.F. (2025). Phonon-boundary scattering and second-order boundary conditions: Application to the heat transfer in one-dimensional nanosystems. J. Therm. Stresses.

[22] Lebon G., Jou D., Dauby P.C. (2012). Beyond the Fourier heat conduction law and the thermal non-slip condition. Phys. Lett. A.

[23] Xu M. (2014). Slip boundary condition of heat flux in Knudsen layers. Proc. R. Soc. A.

[24] Hua Y.-C., Cao B.-Y. (2017). Slip Boundary Conditions in Ballistic-Diffusive Heat Transport in Nanostructures. Nanoscale Microscale Thermophys. Eng..

[25] Xu M. (2021). Phonon hydrodynamic transport and Knudsen minimum in thin graphite. Phys. Lett. A.

[26] Xu M. (2023). Boundary effect and heat vortices of hydrodynamic heat conduction in graphene. Phys. Lett. A.

[27] Prigogine I. (1961). Introduction to Thermodynamics of Irreversible Processes.

[28] Verhás J. (1997). Thermodynamics and Rheology.

[29] Smith G.F. (1971). On isotropic functions of symmetric tensors, skew-symmetric tensors and vectors. Int. J. Engng. Sci..

[30] Di Domenico M., Jou D., Sellitto A. (2020). Nonlinear heat waves and some analogies with nonlinear optics. Int. J. Heat Mass Transf..

[31] Guyer R.A., Krumhansl J.A. (1966). Solution of the linearized phonon Boltzmann equation. Phys. Rev..

[32] Xu M. (2024). The Modified Guyer-Krumhansl Equations Derived From the Linear Boltzmann Transport Equation. J. Heat Mass Transf. ASME.

[33] Xu M. (2022). Effect of inflow boundary conditions on phonon transport in suspended graphene. Phys. Lett. A.

[34] Sellitto A., Carlomagno I., Jou D. (2015). Two-dimensional phonon hydrodynamics in narrow strips. Proc. R. Soc. A.

[35] Ferry D.K., Goodnick S.M. (2009). Transport in Nanostructures.

[36] Luo T., Chen G. (2013). Nanoscale heat transfer—From computation to experiment. Phys. Chem. Chem. Phys..

[37] Asheghi M., Leung Y.K., Wong S.S., Goodson K.E. (1997). Phonon-boundary scattering in thin silicon layers. Appl. Phys. Lett..

[38] Li D., Wu Y., Fan R., Yang P., Majumdar A. (2003). Thermal conductivity of Si/SiGe superlattice nanowires. Appl. Phys. Lett..

[39] Liu W., Asheghi M. (2004). Phonon-boundary scattering in ultrathin single-crystal silicon layers. Appl. Phys. Lett..

